# GSK3β Is Involved in the Relief of Mitochondria Pausing in a Tau-Dependent Manner

**DOI:** 10.1371/journal.pone.0027686

**Published:** 2011-11-14

**Authors:** María Llorens-Martín, Guillermo López-Doménech, Eduardo Soriano, Jesús Avila

**Affiliations:** 1 Departmento de Neurobiología Molecular, Centro de Biología Molecular Severo Ochoa, Madrid, Spain; 2 Centro de Investigación Biomédica en Red sobre Enfermedades Neurodegenerativas, Madrid, Spain; 3 Developmental Neurobiology and Regeneration, Institut for Research in Biomedicine, Barcelona, Spain; 4 Department of Cell Biology, University of Barcelona, Barcelona, Spain; McGill University, Canada

## Abstract

Mitochondrial trafficking deficits have been implicated in the pathogenesis of several neurological diseases, including Alzheimer's disease (AD). The Ser/Thre kinase GSK3β is believed to play a fundamental role in AD pathogenesis. Given that GSK3β substrates include Tau protein, here we studied the impact of GSK3β on mitochondrial trafficking and its dependence on Tau protein. Overexpression of GSK3β in neurons resulted in an increase in motile mitochondria, whereas a decrease in the activity of this kinase produced an increase in mitochondria pausing. These effects were dependent on Tau proteins, as Tau (−/−) neurons did not respond to distinct GSK3β levels. Furthermore, differences in GSK3β expression did not affect other parameters like mitochondria velocity or mitochondria run length. We conclude that GSK3B activity regulates mitochondrial axonal trafficking largely in a Tau-dependent manner.

## Introduction

Axonal transport is essential for neuron function and viability. The axon is mostly devoid of the machinery for the synthesis of proteins and lipids and for the biogenesis of most organelles. Thus efficient axonal transport is required to supply these components and to move organelles from the cell body to the axonal distal region. Several neurodegenerative diseases, like amyotrophic lateral sclerosis, Huntington's disease, Charcot-Marie-Tooth disease, and Alzheimer's disease, exhibit axonal transport defects [1]. In Huntington's disease, pathogenic huntingtin inhibits axonal transport by activating a kinase that modifies the motor protein kinesin involved in transport [2], [3]. Also, mutations in molecular motors have been reported in Charcot-Marie-Tooth disease [3].

In Alzheimer's disease (AD), beta amyloid peptide (Aβ) alters axonal transport [4], [5], [6] through a mechanism involving kinase activation and modification of proteins that may regulate axonal transport [7]. Moreover, Tau protein is required for Aβ-induced defects in axonal transport [8].

Several kinases and substrates may be involved in axonal transport. For example, CK1 activates minus-end-directed transport of organelles along microtubules [9]. Another kinase that may participate in plus-end-directed transport in axons is GSK3. In AD, Aβ peptide binds to Wnt [10], insulin [11] and NMDA [12], [13] receptors, all of which promote an increase in GSK3 activity, and this kinase phosphorylates kinesin-1, thereby impairing transport [14]. However, the subsequent GSK3 activation may promote tau phosphorylation, thereby preventing its interaction with microtubules or facilitating its interaction with kinesin-1 [15]. The participation of tau in axonal transport has been previously described, indicating that it interferes with the binding of motor proteins to microtubules [16]. Also, tau protein is required for Aβ-induced defects in axonal transport [8]. However, little is known about the contribution of phosphotau to this transport. It has been suggested that tau modified by kinases other than GSK3 detaches from microtubules to facilitate organelle transport [17]. Here we studied the participation of GSK3β in mitochondrial trafficking and the dependence of the effects observed on tau protein, a GSK3β substrate. Our results indicate that GSK3 activity increases the number of mitochondria transported through the axon in a tau-dependent manner.

## Materials and Methods

### Neuronal primary culture and transfection

E16 mouse brains were dissected in PBS containing 0.6% glucose and the hippocampi were obtained. After trypsin (Invitrogen, Carlsbad, CA) and DNase treatment (Roche Diagnostics), tissue pieces were dissociated by gentle sweeping. Cells were then counted and seeded onto 0.5 mg/ml poly-L-lysine (Sigma-Aldrich)-coated coverslips (for immunocytochemistry) or 35-mm Fluorodish plates (World Precision Instruments, Inc) for live-imaging in neurobasal medium (Gibco) containing 2 mM glutamax, 120 µg/ml Penicillin, 200 µg/ml Streptomycin and B27 supplement (Invitrogen, Carlsbad, CA), and were maintained at 37°C in the presence of 5% CO2. Cells were cultured for 7 days. Neuronal transfection was carried out at 4 DIV using Lipofectamine 2000 (Invitrogen) following the manufacturer's instructions and using a 1∶3 DNA ratio when transfection of two constructs was required. Cells were processed 24–48 h after transfection.

#### Animal care

Mice were obtained from the *Centro de Biologıía Molecular* “Severo Ochoa” and treated following the guidelines of Council of Europe Convention ETS123, recently revised as indicated in the Directive 86/609/EEC. Animal experiments were performed under protocols (P22/P23) approved by the Institutional Animal Care and Utilization Committee of the *Centro de Biología Molecular Severo Ochoa* (CEEA-CBM, approval certificate number SAF2006-02424 issued on October 10, 2006), Madrid, Spain.

### Live-imaging and quantification of axonal transport of mitochondria

Hippocampal neurons were seeded onto poly-L-lysine-coated Fluorodish plates (World Precision Instruments, Inc), transfected with either GSK3β wt-Myc [18], GSK3β negative dominant-Myc (GSK3βK85) [19], or MitDsRed, and filmed 24 to 48 h after using a Leica TCS SP2 confocal microscope (Leica Microsystems) equipped with a 63× immersion oil objective. All the cultures were kept at 37°C using a heating insert on the microscope stage and an incubating chamber allowing circulation of a controlled CO_2_ (5%)-air heated mixture for the control of pH. Movies were generated at 10 frames per second. For measurements of axonoplasmic transport of mitochondria, axonal processes in transfected neurons were identified following morphological criteria, and directionality was determined for each axon. Axonal mitochondria were registered with an additional digital zoom of 1.7×. Time-lapse series of image stacks composed of 5 images (512×512 px) were taken every 6 sec during 15 min. All 151 images obtained were processed mainly with Leica Confocal Software. Further image processing, analysis and video compilation (10 frames per second) and edition was done with ImageJ software (version 1.43K, NIH, USA). Kymographs were generated with MetaMorph Software (Molecular Devices - MDS Analytical Technologies). In overexpression experiments, a minimum of 10 axons were analyzed in each condition. The axons recorded were selected on the basis of detection of MitDsRed fluorescence. After live-imaging recording, cultures were fixed and immunostained against Myc-tag in order to ensure that MitDsRed-transfected axons were effectively co-transfected with GSK3β. Only double-transfected axons were analyzed. More than 80% of axons transfected with MitDsRed were also co-transfected with either GSK3β wt or DN-GSK3β. In all cases a mitochondrion was considered motile when it moved more than 0.5 µm during 1 min of recording. Distances and speeds of retrograde and anterograde transport were measured separately from the corresponding kymographs, as previously described [20], and no tracking-plugging was used. The first frame recorded of each video was digitally processed and thresholded (using Yi algorithm). The longest distance between any two points along the selection boundary (Feret's parameter) was then measured for all mitochondria. To ensure equal relevance between axons, independently of the number of mitochondria displayed, an average length was calculated for each neuron and then the mean was calculated between axons.

### Immunocytochemistry

For Phospho-Tau and apoptotic marker detection, double immunocytochemistry was performed as described previously [18] by incubating the cells with the following primary antibodies for 2 h: mouse anti-hyperphosphorylated Tau (PHF-1) (1∶1000, a kind gift from Dr. Peter Davis, New York , USA), rabbit-anti cleaved-caspase 3 (Cell Signaling Technology, Massachusetts, 1∶2,000), rabbit anti-fractin (Becton Dickinson, NJ, USA, 1∶2,000) and rabbit anti-myc (Abcam, 1∶2000). The binding of these antibodies was then detected over 2 h with the following donkey Alexa-conjugated secondary antibodies as appropriate (1∶1000, Molecular Probes, Eugene, OR, US): Anti-rabbit Alexa 647-conjugated, Anti-mouse Alexa 647-conjugated, Anti-rabbit Alexa 488-conjugated and Anti-mouse Alexa 488-conjugated. Cells were counterstained for 10 min with DAPI (1∶10000, Calbiochem-EMD Darmstadt, Germany). Stacks of images were obtained in a Zeiss LSM710 confocal microscope. Stacks were composed by series of images, each one separated by 0.1 µm (63× Oil objective, Digital Zoom 2). Fluorescence intensity along the axonal process was then measured using ImageJ software and then divided by the total axonal length in each stack.

### Statistical Analysis

Data were analyzed conducting a one-way ANOVA test for comparisons between experimental conditions. SPSS 17.0.1 software (SPSS, 1989; Apache Software Foundation) was used for all the statistical analyses.

## Results

### GSK3β overexpression increases mitochondrial axonal transport

**Figure 1:**
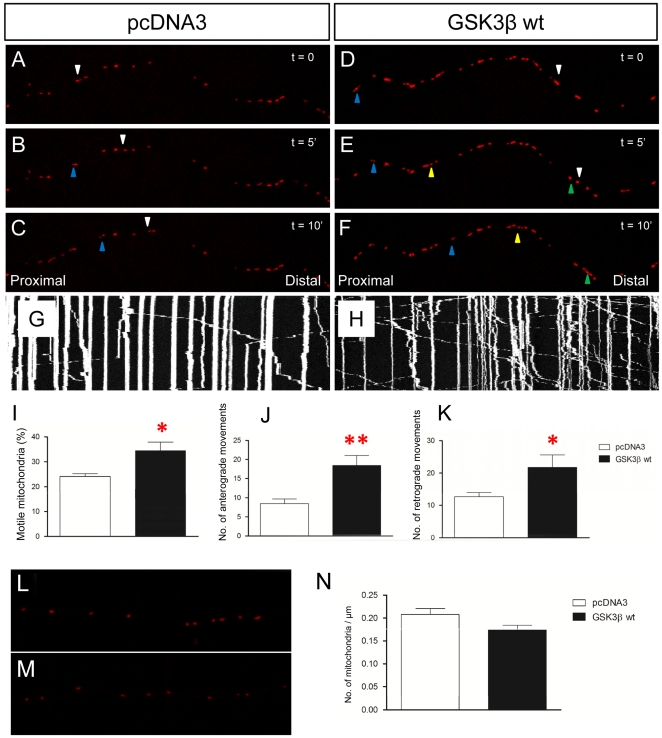
GSK3β overexpression increases the percentage of motile mitochondria. **A–F:** Representative images of primary cultured neuron axons showing the movement of some mitochondria. Motile mitochondria are indicated with colored triangles. Time interval between photographs is 10 seconds. GSK3β wt transfection increases the percentage of motile mitochondria (**D–F**) compared to pcDNA3 transfection (F_1,18_ = 4.117; p = 0.046) (**A–C**). Blue, yellow and green triangles label different individual motile mitochondria respectively. **G–H:** Representative kymographs of pcDNA3- (**G**), GSK3β wt- (**H**) transfected axons respectively. **I:** Quantification of the percentage of motile mitochondria. **J–K:** Number of anterograde (**J**) and retrograde (**K**) movements is increased after GSK3β transfection (Anterograde: F_1,20_ = 9.398; p = 0.006) (Retrograde: F_1,20_ = 4.114; p = 0.049). **L–N:** The number of mitochondria was quantified in several axonal segments and normalized by dividing the number of mitochondria by the length of the axon. **L–M:** Representative images of MitDsRed in pcDNA3 (**L**), GSK3β wt (**M**) transfected primary cultured neurons. N: Quantification of the number of mitochondria per axonal length unit. Red asterisk: * 0.05>p≥0.01; ** 0.01>p≥0.001.

**Figure 2:**
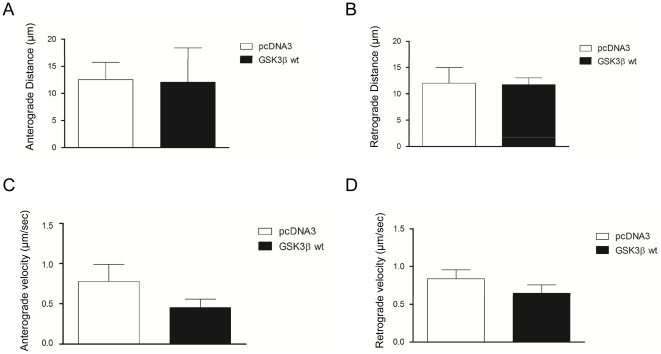
Run length and velocity after GSK3β transfection. Anterograde (**A**) and retrograde (**B**) run length after GSK3β wt transfection. **C–D:** Anterograde (**C**) and retrograde (**D**) velocity of mitochondrial transport after GSK3β wt transfection.

To study the impact of GSK3β on mitochondrial axonal trafficking, we overexpressed GSK3β in cultured hippocampal neurons. Transfected neurons were video-recorded 6–7 days after transfection (Figure 1). Overexpression of GSK3β did not alter the density of axonal mitochondria (F_2,29_ = 2.591; p = 0.093) (Figure 1 L–M). In agreement with previous studies, 15-min time-lapse recordings of control neurons (transfected with the empty vector) revealed that that most mitochondria remained stationary, while about 25% moved actively within the axons. GSK3β wt transfection increased the percentage of motile mitochondria (Figure 1 I) [21]. In an additional characterization, the number of anterograde and retrograde movements were measured for each sample (Figure 1 J and 1 K, respectively). The marked increase in motile mitochondria after GSK3β overexpression accounts for both the anterograde and retrograde directions (Figure 1 J and 1 K, respectively). Finally, GSK3β overexpression did not significantly alter the distance travelled by single motile mitochondria (Figure 2 A and 2 B) or their velocity (Figure 2 C and 2 D), in either the anterograde (Figure 2 A and 2 C) or retrograde (Figure 2 B and 2 D) directions. We thus conclude that GSK3 overexpression essentially leads to an increase in the number of motile mitochondria.

### GSK3β kinase activity regulates the number of transported mitochondria and their velocity

**Figure 3:**
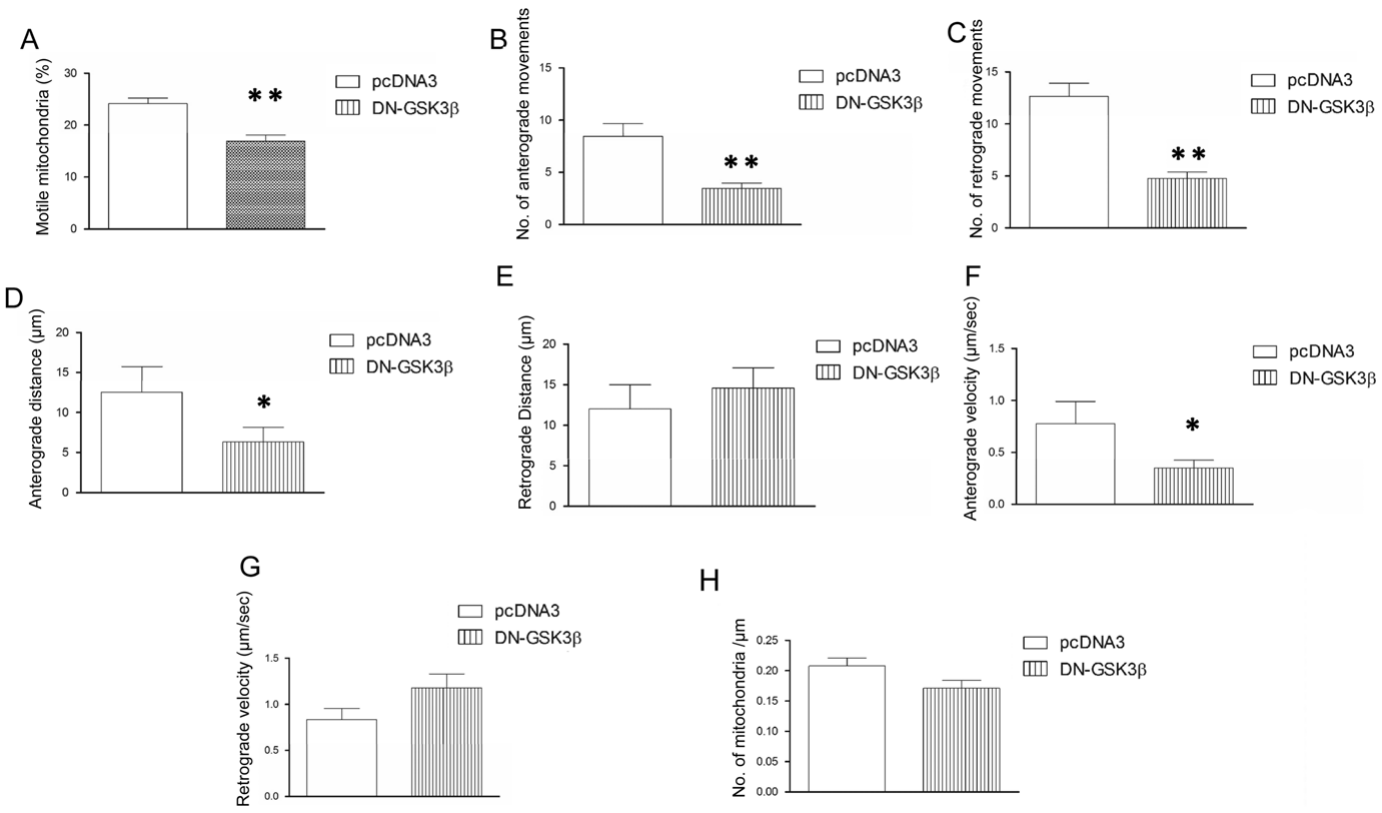
Effects of the absence of GSK3β activity. **A:** Dominant negative GSK3β K85 (DN- GSK3β) transfection reduces the percentage of motile mitochondria (F_1,16_ = 16.794; p = 0.001). **B:** Number of anterograde movements after DN-GSK3β transfection is increased compared to pcDNA3-transfected neurons (F_1,16_ = 10.085; p = 0.007). **C:** Number of retrograde movements after DN-GSK3β transfection is decreased compared to that of pcDNA3-transfected neurons (F_1,17_ = 32.054; p = 0.001). **D–E:** Absence of GSK3β activity modifies the average anterograde (F_1,18_ = 5.297; p = 0.034) but not the retrograde run length. **F–G:** The lack of GSK3β activity decreases the velocity of anterograde mitochondrial trafficking (**F**) (F_1,17_ = 4.257; p = 0.048) whereas it does not modify the speed of individual retrograde movements (**G**). **H:** The number of mitochondria per length unit remains unchanged after DN-GSK3β transfection. Asterisk: * 0.05>p≥0.01; ** 0.01>p≥0.001.

**Figure 4:**
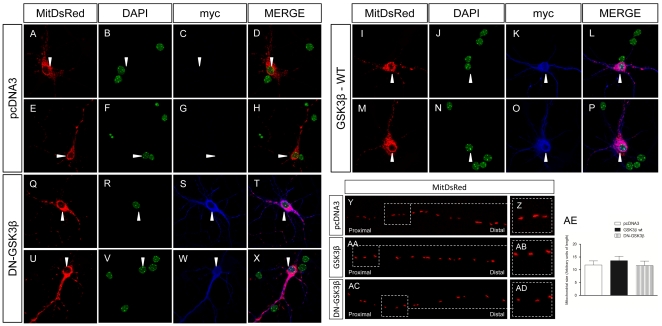
Cell morphology and appearance does not change after transfection with -GSK3β wt or DN-GSK3β. **A–X:** The general morphology, nuclear chromatin condensation, neurite number and appearance remains unchanged after co-transfection of MitDsRed and pcDNA3 (**A**–**H**), GSK3β wt (**I**–**P**) or DN-GSK3β (**Q–X**). MitDsRed: red; DAPI: Green; myc: Blue. White triangles label the soma of each individual tranfected neuron. **Y-AE:** Mitochondrial morphology, roundness and density do not change after transfection with GSK3β wt (**AA**–**AB**) or DN-GSK3β (**AC**–**AD**) compared to pcDNA3-transfected neurons (**Y**–**Z**). In **Z**, **AB** and **AD** high magnification of several mitochondria represented respectively in **Y, AA** and **AC** can be observed. MitDsRed: Red. **AE**: Quantification of mitochondrial size.

To study whether GSK3β kinase activity is required for the above effects, we transfected neurons with a construct expressing a dominant-negative GSK3β variant lacking kinase activity (DN-GSK3β) [19]. Overexpression of DN-GSK3β did not alter the density of axonal mitochondria (Figure 3 H). The GSK3β-mediated increase in mitochondrial motility was not observed when neurons were transfected with the DN-GSK3β variant. Indeed, opposite effects were found (Figure 3). Again, this effect accounted for both the anterograde (Figure 3 B, 3 D and 3 F) and retrograde (Figure 3 C, 3 E and 3 G) directions of transport. Moreover, transfection with DN-GSK3β resulted in the opposite effects, thereby leading to a marked reduction in the number of motile mitochondria compared to control neurons (Figure 3 A). In addition, the number of anterograde (Figure 3 B) and retrograde (Figure 3 C) movements was reduced due to DN-GSK3β transfection. Similarly, the absence of GSK3β activity modified the average anterograde but not the retrograde run length travelled by individual mitochondria (Figure 3 D and 3 E respectively). Finally, the lack of GSK3β activity decreased the velocity of anterograde mitochondrial trafficking (Figure 3 F) but did not modify the speed of individual retrograde movements (Figure 3 G). Taken together, these findings suggest that GSK3β activity regulates not only the number of motile mitochondria through the axon but also the trafficking speed of these mitochondria. Finally, the absence of GSK3β activity did not modify the general morphology or appearance of the transfected neurons (Figure 4). Similarly, axonal mitochondria showed similar densities (Figure 3 H), morphology, size, roundness and absence of aggregation in all the experimental conditions tested (Figure 4 Y- AE). Similarly, general morphology, nuclear chromatin condensation, neurite number and appearance remained unchanged after co-transfection of MitDsRed and pcDNA3 (Figure 4 A–H), GSK3β wt (I–P) or DN-GSK3β (Q–X). In line with this, DN-GSK3β transfection did not induce apoptotic processes, as can be observed in Supplementary Figure S1.

### Tau protein is required for the effects of GSK3β overexpression on mitochondrial trafficking

**Figure 5:**
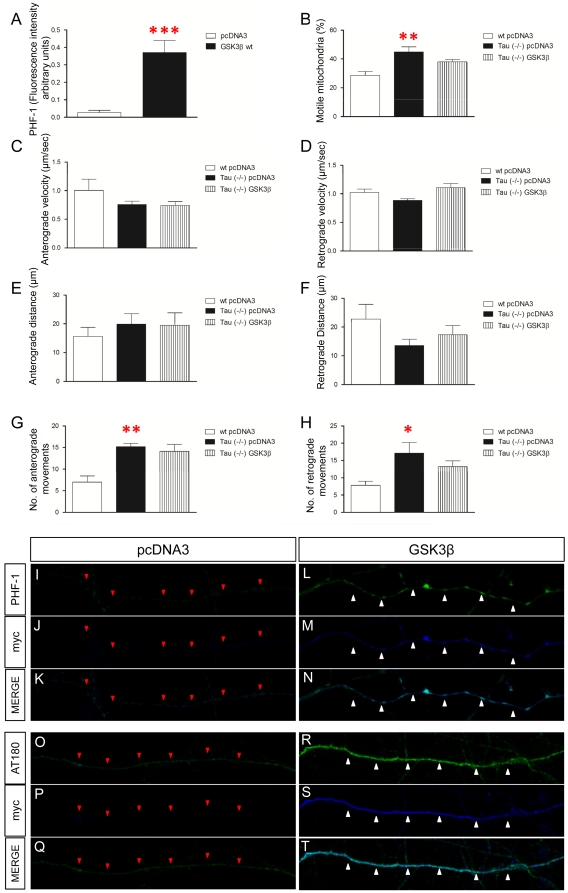
GSK3β overexpression effects are mediated by Tau phosphorylation. **A:** Tau phosphorylation is increased after GSK3β overexpression (F_1,18_ = 20.987; p = 0.0001). **B:** Percentage of motile mitochondria in Tau (−/−) neurons is increased compared to wt (F_1,26_ = 9.34; p = 0.001) whereas GSK3β overexpression in Tau (−/−) neurons does not modify this parameter. **C–D:** Anterograde (**C**) or retrograde (**D**) mitochondrial velocity in the absence of tau and after overexpression of GSK3β. **E–H:** Tau absence does not modify mitochondria run length but increases the number of anterograde and retrograde movements. **E–F:** Neither anterograde (**E**) nor retrograde (**F**) run length is modified in the absence of Tau or after overexpression of GSK3β. **G–H:** Number of anterograde (**G**) and retrograde (**H**) movements in the absence of tau and after overexpression of GSK3β in Tau (−/−) neurons. Tau absence increases the number of both anterograde (F_1,26_ = 9.164; p = 0.001) and retrograde movements (F_1,28_ = 4.992; p = 0.015) compared to wt neurons. **I–T:** Phosphorylation of tau is increased after GSK3β transfection. PHF-1 staining in GSK3β- (**L**–**N**) and pcDNA3-transfected axons (**I**–**K**). PHF-1: Green; myc: Blue. Similarly, AT180 staining is also increased in GSK3β- (**R**–**T**) compared to pcDNA3-transfected axons (**O**–**Q**). AT180: Green; myc: Blue. Red triangles indicate pcDNA3-transfected axons. White triangles label GSK3β-transfected axons. Red asterisk: * 0.05>p>0.01; ** 0.01>p≥0.001; *** 0.001>p≥0.0001.

Axonal mitochondrial transport requires the association between microtubules and mitochondria [22]. Two protein superfamilies, the kinesins and dyneins, serve as molecular motors for anterograde or retrograde transport of mitochondria, respectively. The binding of these proteins to microtubules is regulated by microtubule-associated proteins (MAPs), such as tau [23] and MAP1B [24]. In fact, it has been proposed that the presence of MAPs increases mitochondria pausing [24]. Tau phosphorylation by GSK3 could result in a decrease in the interaction of this MAP with microtubules [21], [25] and it may increase the binding of motors to microtubules and the number of motile mitochondria. To test whether Tau, a GSK3 substrate, is involved in the effects described above, we first analyzed the levels of Tau phosphorylation after GSK3β overexpression, by using PHF-1 and AT180 antibodies [18] (Figure 5). A dramatic increase in tau phosphorylation was detected when GSK3β was overexpressed (Figure 5 A). Representative images of AT180 (Figure 5 O–T) and PHF-1 (Figure 5 I–O) hyperphosphorylated tau in pcDNA3- and GSK3β-transfected neurons can be observed in Figure 5. Thus, we examined the contribution of GSK3β to mitochondria transport in the absence of tau. For this purpose, we transfected the GSK3β expression vector in Tau (−/−) neurons and wild-type (WT) littermate controls. In the absence of tau, an increase in the number of motile mitochondria was observed in control neurons (Figure 5 B). Moreover, in neurons lacking tau protein, GSK3β overexpression did not result in changes in any of the mitochondrial trafficking parameters measured (Figure 5 B–F), in contrast to WT neurons overexpressing GSK3β (see Figure 3). Thus, similar values for mitochondria run length (Figure 5 E, 5 F), anterograde (Figure 5 C) and retrograde (Figure 5 D) velocities, and number of anterograde (Figure 5 G) or retrograde (Figure 5 H) movements were found in the absence and in the presence of GSK3 overexpression in Tau-deficient neurons. These data strongly suggest that the effects of GSK3β on mitochondrial trafficking are largely mediated by the MAP protein Tau.

Thus, an increase in the number of motile mitochondria was found in the absence of tau protein (Figure 5 B). This effect was similar to that observed upon an increase in GSK3β activity (Figure 1 I). Furthermore, in the absence of tau, no differences in axonal transport rates were found, as previously reported [26]. In contrast, in the presence of increased GSK3β activity, we detected differences in the retrograde trafficking velocity of mitochondria. In this regard, proteins other than tau could be involved in the GSK3β-dependent effect on retrograde axonal mitochondria transport.

## Discussion

Here we found that overexpression of GSK3β in cultured neurons results in an increase in the number of axonal motile mitochondria. About two thirds of the total axonal mitochondria remained stationary in our neuronal cultures but this number markedly decreased when GSK3β was overexpressed. There is no clear explanation for the possible function of mitochondria pausing. Axonal transport of healthy mitochondria is required to carry them to the growth cones, or to the synapses, where the presence of these organelles is required [27], [28] It has been suggested that when growth cone activity is low, mitochondria switch to retrograde movement [27]. Also, dysfunctional mitochondria are retrogradely transported towards the cell body to undergo repair, a process that occurs by fusion with healthy mitochondria or degradation by mitophagy [29], [30], [31]. Thus, an increase in stationary mitochondria may result in the lack of all, or some, of these functions. Moreover, mitochondrial pausing has been related to enhanced neurotransmission and Ca2^+^ elevations, thereby supplying the energy required for local excitability events [32], [33]. Here we demonstrate that the expression of GSK3β facilitates the movement of mitochondria. On the basis of this observation, we propose that GSK3 levels and activity modulate some of the crucial events mentioned above, as previously demonstrated [34].

Here we found that the effects of GSK3 overexpression on mitochondrial trafficking were abolished in Tau (−/−) neurons, thereby indicating that the effects of this kinase are mediated by its action on the modification of Tau. Moreover, it is well known that MAPs like tau protein share and compete with microtubule motors for the same binding sites to microtubules [35], [36], [37]. Indeed, we found that GSK3β overexpression results in an increase in tau phosphorylation and a decrease of tau bound to microtubules.

We also observed some differences in anterograde (decrease) and retrograde (increase) rates when GSK3β activity was decreased with the DN-GSK3 construct, which also acts as a dominant negative [19], [38]. In this way, it has recently been demonstrated that mitochondrial axonal transport is specifically inhibited by treatment with the GSK3 inhibitor lithium [39]. There are several possible explanations for these findings. Tau interferes with the binding of motor proteins, like kinesin, to microtubules [16], [23], [40] and there is a gradient of tau along the axon, with the highest levels found at the distal part of the axon [41], which could explain the detachment of kinesin close to the synapses. This observation might explain the fall in anterograde velocity observed when GSK3β activity decreased. Moreover, Tau protein not only competes with molecular motors for binding to microtubules [23] but it has also been suggested that tau inhibits kinesin activity [16], [35], [42], through a mechanism involving tau phosphorylation [43].

A similar gradient has been found for GSK3-phosphorylated MAP1B [44]. Although the functional consequences of this phosphorylation are unknown, some modes of phosphorylation may favor the binding of MAP1B to microtubules [45], [46]. Furthermore, a higher competition of MAP1B with dynein for microtubule binding sites has been proposed [24]. Thus, the alterations in retrograde mitochondrial transport reported here could be mediated by changes in the phosphorylation of MAP1B induced by GSK3β.

In summary, here we have shown that GSK3β activity participates in the relief mitochondrial pausing, and that this relief depends largely on Tau proteins. In addition, we demonstrate that an increase in GSK3 activity results in differences in trafficking velocities of axonal mitochondria, which we propose to be dependent on both Tau and other MAP proteins.

## Supporting Information

Figure S1 **Transfection with DN-GSK3β does not induce apoptotic processes.** **A–O:** Immunocytochemistry against Caspase 3. DN-GSK3β transfection does not induce apoptosis. As can be seen in the images, apoptotic cells (white asterisk) can be observed among the field, but no-colocalization with MitDsRed or with myc can be appreciated. A double transfected neuron is labeled with a white arrow. **A–E** and **K–O:** High magnification images of the squared area in **F–J**. **P–Y:** Immunocytochemistry against Fractin (Caspase-3 cleaved fragment of actin). Apoptotic cells (white asterisk) can be observed among the field, but no colocalization with MitDsRed or with myc can be appreciated. A double transfected neuron is labeled with a white arrow. **U–Y**: High magnification images of the squared area in **P–T**.
(TIF)

## References

[1] Morfini GA, Burns M, Binder LI, Kanaan NM, LaPointe N (2009). Axonal transport defects in neurodegenerative diseases.. J Neurosci.

[2] Morfini GA, You YM, Pollema SL, Kaminska A, Liu K (2009). Pathogenic huntingtin inhibits fast axonal transport by activating JNK3 and phosphorylating kinesin.. Nat Neurosci.

[3] Berciano J (2011). Peripheral neuropathies: Molecular diagnosis of Charcot-Marie-Tooth disease.. Nat Rev Neurol.

[4] Hiruma H, Katakura T, Takahashi S, Ichikawa T, Kawakami T (2003). Glutamate and amyloid beta-protein rapidly inhibit fast axonal transport in cultured rat hippocampal neurons by different mechanisms.. J Neurosci.

[5] Pigino G, Morfini G, Atagi Y, Deshpande A, Yu C (2009). Disruption of fast axonal transport is a pathogenic mechanism for intraneuronal amyloid beta.. Proc Natl Acad Sci U S A.

[6] Shah SB, Nolan R, Davis E, Stokin GB, Niesman I (2009). Examination of potential mechanisms of amyloid-induced defects in neuronal transport.. Neurobiol Dis.

[7] Decker H, Lo KY, Unger SM, Ferreira ST, Silverman MA (2010). Amyloid-beta peptide oligomers disrupt axonal transport through an NMDA receptor-dependent mechanism that is mediated by glycogen synthase kinase 3beta in primary cultured hippocampal neurons.. J Neurosci.

[8] Vossel KA, Zhang K, Brodbeck J, Daub AC, Sharma P (2010). Tau reduction prevents Abeta-induced defects in axonal transport.. Science.

[9] Ikeda K, Zhapparova O, Brodsky I, Semenova I, Tirnauer JS (2011). CK1 activates minus-end-directed transport of membrane organelles along microtubules.. Mol Biol Cell.

[10] Magdesian MH, Carvalho MM, Mendes FA, Saraiva LM, Juliano MA (2008). Amyloid-beta binds to the extracellular cysteine-rich domain of Frizzled and inhibits Wnt/beta-catenin signaling.. J Biol Chem.

[11] Townsend M, Mehta T, Selkoe DJ (2007). Soluble Abeta inhibits specific signal transduction cascades common to the insulin receptor pathway.. J Biol Chem.

[12] Hoshi M, Sato M, Matsumoto S, Noguchi A, Yasutake K (2003). Spherical aggregates of beta-amyloid (amylospheroid) show high neurotoxicity and activate tau protein kinase I/glycogen synthase kinase-3beta.. Proc Natl Acad Sci U S A.

[13] De Felice FG, Wu D, Lambert MP, Fernandez SJ, Velasco PT (2008). Alzheimer's disease-type neuronal tau hyperphosphorylation induced by A beta oligomers.. Neurobiol Aging.

[14] Morfini G, Szebenyi G, Elluru R, Ratner N, Brady ST (2002). Glycogen synthase kinase 3 phosphorylates kinesin light chains and negatively regulates kinesin-based motility.. EMBO J.

[15] Cuchillo-Ibanez I, Seereeram A, Byers HL, Leung KY, Ward MA (2008). Phosphorylation of tau regulates its axonal transport by controlling its binding to kinesin.. FASEB J.

[16] Ebneth A, Godemann R, Stamer K, Illenberger S, Trinczek B (1998). Overexpression of tau protein inhibits kinesin-dependent trafficking of vesicles, mitochondria, and endoplasmic reticulum: implications for Alzheimer's disease.. J Cell Biol.

[17] Mandelkow EM, Thies E, Trinczek B, Biernat J, Mandelkow E (2004). MARK/PAR1 kinase is a regulator of microtubule-dependent transport in axons.. J Cell Biol.

[18] Lucas JJ, Hernandez F, Gomez-Ramos P, Moran MA, Hen R (2001). Decreased nuclear beta-catenin, tau hyperphosphorylation and neurodegeneration in GSK-3beta conditional transgenic mice.. EMBO J.

[19] Dominguez I, Itoh K, Sokol SY (1995). Role of glycogen synthase kinase 3 beta as a negative regulator of dorsoventral axis formation in Xenopus embryos.. Proc Natl Acad Sci U S A.

[20] De Vos KJ, Sheetz MP (2007). Visualization and quantification of mitochondrial dynamics in living animal cells.. Methods Cell Biol.

[21] Trinczek B, Biernat J, Baumann K, Mandelkow EM, Mandelkow E (1995). Domains of tau protein, differential phosphorylation, and dynamic instability of microtubules.. Mol Biol Cell.

[22] Frederick RL, Shaw JM (2007). Moving mitochondria: establishing distribution of an essential organelle.. Traffic.

[23] Dixit R, Ross JL, Goldman YE, Holzbaur EL (2008). Differential regulation of dynein and kinesin motor proteins by tau.. Science.

[24] Jimenez-Mateos EM, Gonzalez-Billault C, Dawson HN, Vitek MP, Avila J (2006). Role of MAP1B in axonal retrograde transport of mitochondria.. Biochem J.

[25] Utton MA, Vandecandelaere A, Wagner U, Reynolds CH, Gibb GM (1997). Phosphorylation of tau by glycogen synthase kinase 3beta affects the ability of tau to promote microtubule self-assembly.. Biochem J.

[26] Yuan A, Kumar A, Peterhoff C, Duff K, Nixon RA (2008). Axonal transport rates in vivo are unaffected by tau deletion or overexpression in mice.. J Neurosci.

[27] Morris RL, Hollenbeck PJ (1995). Axonal transport of mitochondria along microtubules and F-actin in living vertebrate neurons.. J Cell Biol.

[28] Mackey S, Schuessler G, Goldberg DJ, Schwartz JH (1981). Dependence of fast axonal transport on the local concentration of organelles.. Biophys J.

[29] Hollenbeck PJ, Saxton WM (2005). The axonal transport of mitochondria.. J Cell Sci.

[30] Chang DT, Reynolds IJ (2006). Mitochondrial trafficking and morphology in healthy and injured neurons.. Prog Neurobiol.

[31] Anesti V, Scorrano L (2006). The relationship between mitochondrial shape and function and the cytoskeleton.. Biochim Biophys Acta.

[32] MacAskill AF, Atkin TA, Kittler JT (2010). Mitochondrial trafficking and the provision of energy and calcium buffering at excitatory synapses.. Eur J Neurosci.

[33] MacAskill AF, Kittler JT (2010). Control of mitochondrial transport and localization in neurons.. Trends Cell Biol.

[34] Morel M, Authelet M, Dedecker R, Brion JP (2010). Glycogen synthase kinase-3beta and the p25 activator of cyclin dependent kinase 5 increase pausing of mitochondria in neurons.. Neuroscience.

[35] Seitz A, Kojima H, Oiwa K, Mandelkow EM, Song YH (2002). Single-molecule investigation of the interference between kinesin, tau and MAP2c.. EMBO J.

[36] Mandelkow EM, Stamer K, Vogel R, Thies E, Mandelkow E (2003). Clogging of axons by tau, inhibition of axonal traffic and starvation of synapses.. Neurobiol Aging.

[37] Stamer K, Vogel R, Thies E, Mandelkow E, Mandelkow EM (2002). Tau blocks traffic of organelles, neurofilaments, and APP vesicles in neurons and enhances oxidative stress.. J Cell Biol.

[38] Gomez-Sintes R, Hernandez F, Bortolozzi A, Artigas F, Avila J (2007). Neuronal apoptosis and reversible motor deficit in dominant-negative GSK-3 conditional transgenic mice.. EMBO J.

[39] Zhang LF, Shi L, Liu H, Meng FT, Liu YJ (2011). Increased hippocampal tau phosphorylation and axonal mitochondrial transport in a mouse model of chronic stress.. Int J Neuropsychopharmacol.

[40] Kanaan NM, Morfini G, Pigino G, Lapointe NE, Andreadis A (2011). Phosphorylation in the amino terminus of tau prevents inhibition of anterograde axonal transport.. Neurobiol Aging.

[41] Mandell JW, Banker GA (1996). A spatial gradient of tau protein phosphorylation in nascent axons.. J Neurosci.

[42] Vershinin M, Carter BC, Razafsky DS, King SJ, Gross SP (2007). Multiple-motor based transport and its regulation by Tau.. Proc Natl Acad Sci U S A.

[43] Kanaan NM, Morfini GA, LaPointe NE, Pigino GF, Patterson KR (2011). Pathogenic forms of tau inhibit kinesin-dependent axonal transport through a mechanism involving activation of axonal phosphotransferases.. J Neurosci.

[44] Avila J, Ulloa L, Diez-Guerra J, Diaz-Nido J (1994). Role of phosphorylated MAPlB in neuritogenesis.. Cell Biol Int.

[45] Diaz-Nido J, Serrano L, Mendez E, Avila J (1988). A casein kinase II-related activity is involved in phosphorylation of microtubule-associated protein MAP-1B during neuroblastoma cell differentiation.. J Cell Biol.

[46] Ulloa L, Diaz-Nido J, Avila J (1993). Depletion of casein kinase II by antisense oligonucleotide prevents neuritogenesis in neuroblastoma cells.. EMBO J.

