# Supplementary figures and images for: GSK3β Is Involved in the Relief of Mitochondria Pausing in a Tau-Dependent Manner
Source: PLoS One. 2011 Nov 14;6(11):e27686. doi: 10.1371/journal.pone.0027686 (PMC3215736; doi:10.1371/journal.pone.0027686)

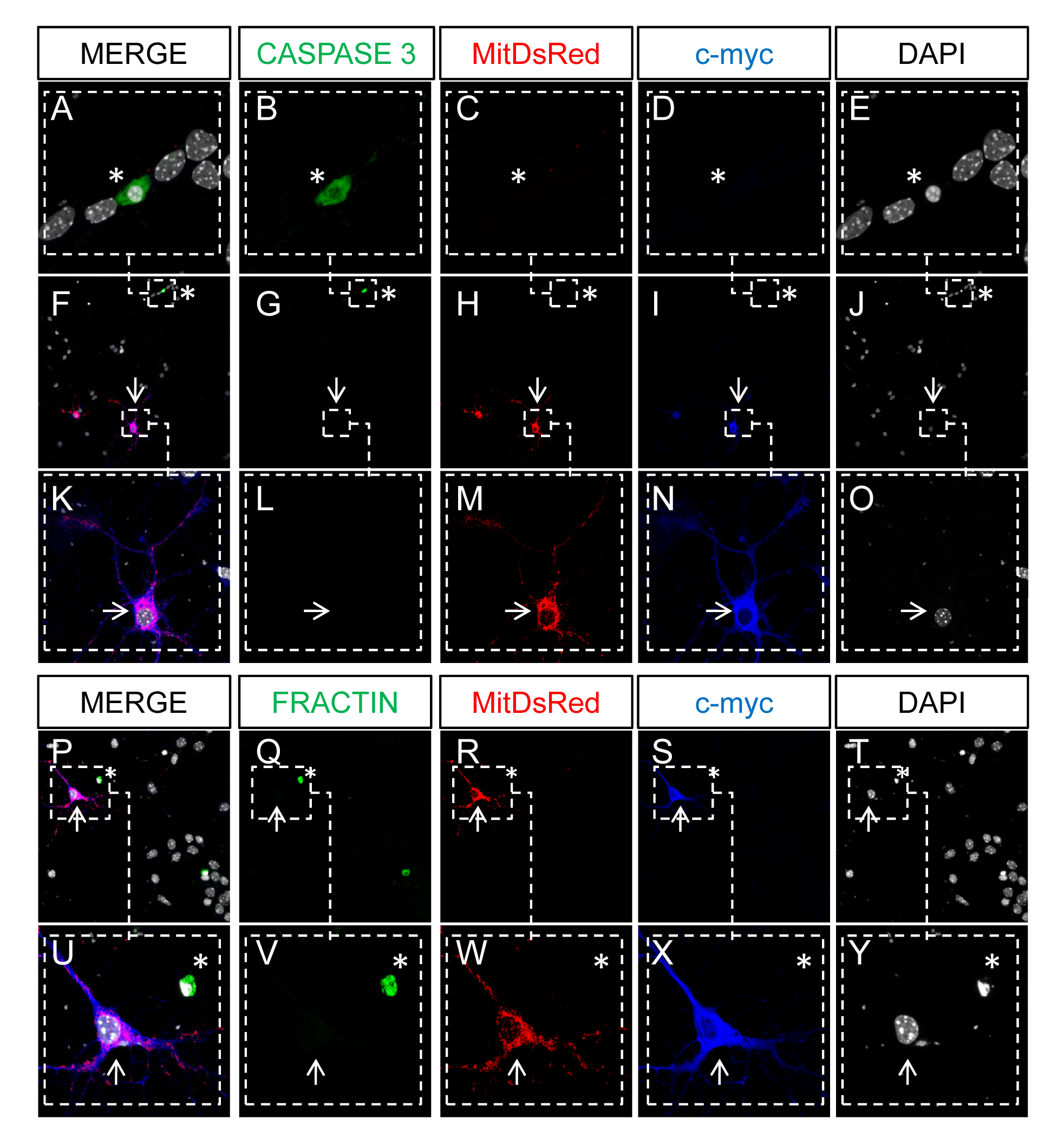

Supplement: Figure S1 — Transfection with DN-GSK3β does not induce apoptotic processes. A–O: Immunocytochemistry against Caspase 3. DN-GSK3β transfection does not induce apoptosis. As can be seen in the images, apoptotic cells (white asterisk) can be observed among the field, but no-colocalization with MitDsRed or with myc can be appreciated. A double transfected neuron is labeled with a white arrow. A–E and K–O: High magnification images of the squared area in F–J. P–Y: Immunocytochemistry against Fractin (Caspase-3 cleaved fragment of actin). Apoptotic cells (white asterisk) can be observed among the field, but no colocalization with MitDsRed or with myc can be appreciated. A double transfected neuron is labeled with a white arrow. U–Y: High magnification images of the squared area in P–T. (TIF) [file pone.0027686.s001.tif]
